# Simpute: An Efficient Solution for Dense Genotypic Data

**DOI:** 10.1155/2013/813912

**Published:** 2013-02-03

**Authors:** Yen-Jen Lin, Chun-Tien Chang, Chuan Yi Tang, Wen-Ping Hsieh

**Affiliations:** ^1^Department of Computer Science, National Tsing Hua University, Hsinchu, Taiwan; ^2^Department of Computer Science and Information Engineering, Providence University, Taichung, Taiwan; ^3^Institute of Statistics, National Tsing Hua University, Hsinchu, Taiwan

## Abstract

Single nucleotide polymorphism (SNP) data derived from array-based technology or massive parallel sequencing are often flawed with missing data. Missing SNPs can bias the results of association analyses. To maximize information usage, imputation is often adopted to compensate for the missing data by filling in the most probable values. To better understand the available tools for this purpose, we compare the imputation performances among BEAGLE, IMPUTE, BIMBAM, SNPMStat, MACH, and PLINK with data generated by randomly masking the genotype data from the International HapMap Phase III project. In addition, we propose a new algorithm called simple imputation (Simpute) that benefits from the high resolution of the SNPs in the array platform. Simpute does not require any reference data. The best feature of Simpute is its computational efficiency with complexity of order (*mw* + *n*), where *n* is the number of missing SNPs, *w* is the number of the positions of the missing SNPs, and *m* is the number of people considered. Simpute is suitable for regular screening of the large-scale SNP genotyping particularly when the sample size is large, and efficiency is a major concern in the analysis.

## 1. Background

A single nucleotide polymorphism (SNP) is a genetic variation at a single base-pair position. It is acquired and retained in the population. Most SNPs produce no observable difference between members of a species. These variations in the DNA can occur on both coding and noncoding sequences at a frequency of approximately 1 per 1000 base pairs [1, 2]. This leads to a rate of an estimated 11 million loci that can vary in approximately 0.1% of the population according to neutral theory of population genetics [3].

Studies concerning genetic association examine genetic traits shared among individuals in a population. SNPs have an important role in these studies because they record the history of recombination and are sufficiently dense to form linkage disequilibrium (LD) in nearly all functional genes. However, it is common for data to be missing on the various genotyping platforms. Even for array technology, the rate of missing data can be as high as 0.53% [4]. This is approximately 5300 loci for every million SNPs designed on the arrays. Assuming a random missing mechanism exists, if any locus in a sample is removed, the missing rate can become as high as 1 − (1−0.0053)^*n*^ in an association study of *n* samples. 

Because it is often not financially viable to regenotype the missing data, imputation is used to fill in the missing SNP values, and to maintain low costs. Imputation can be as simple as selecting at random a genotype that already exists in the data or by using a major allele. However, such naive methods normally result in high error rates [4]. Certain other methods are based on haplotypes, which are sets of SNPs that are associated on one chromosome pair. These methods include the Hidden Markov Model (HMM), Markov chain (MC), maximum-likelihood, and neural network. Because a multitude of methodologies exists that can be employed to impute a haplotype, a range of imputation software, consequently, also exists. Examples of imputation software include IMPUTE [5], MaCH [6], SNPMSTAT [7], fastPhase [8], and BEAGLE [9]. 

Both the IMPUTE and BEAGLE software use the HMM. The HMM is a statistical tool for modelling generative sequences, which are characterised by the use of an underlying process to generate an observable sequence. In the HMM these underlying processes are represented by states, which are considered to be unobserved or hidden. The hidden state used is a pair of haplotypes observed in reference samples from the HapMap project. The observed data are the individual genotypes at the corresponding loci. IMPUTE considers mutation and recombination in its HMM model; this requires additional information from CHIAMO [10] and HAPGEN [6, 10, 11] to determine the probability of the genotypes estimated from the arrays and the predicted haplotypes. MaCH uses a Markov chain-based approach using samples from HapMap as references. Long missing segments are compensated for in MaCH by using haplotypes from the reference samples. 

Alternative imputation software and methodologies include SNPMSTAT and FFNN. SNPMSTAT uses a maximum-likelihood framework on the genotype data. It uses HapMap data or other similar data sets to construct the most-likely haplotypes to occur for a missing SNP value. The feed-forward neural networks (FFNNs) proposed by Sun and Kardia [12] were reported to perform well by using a Bayesian criterion to select the predictors. They claimed that the performance is better than that of fastPHASE [8] and the LD-based method, which is used by HelixTree [10].

In this paper, we propose an algorithm based on observed genotypes and the LD at three neighbouring SNPs, including the SNP under consideration, to impute the missing SNPs, and to reduce the error rate for estimation. This algorithm only considers the two neighbouring SNPs and uses the haplotype information, which is a direct consequence of LD. Jung et al. used the same level of information in their proposed method [4], which phased genotypes by the partition-ligation expectation maximization (PLEM) [11] to impute the missing SNPs. We compare the results using SNPs from the same chromosomal regions in Jung's study and demonstrate the better performance of our algorithm. We also compare the general-purpose methods including BIMBAM v0.99, BEAGLE v3.0.3, IMPUTE v0.5.0, MARCH v1.0.16, PLINK, and SNPMSTAT v3.1. Because Simpute provides the best power at highly linked loci, we compare it to the best method using SNPs with strong LD. We demonstrate that Simpute is a promising tool to provide efficient computation when it comes to the age of massive parallel sequencing.

## 2. Methods

SNPs could be bi-, tri-, or tetraallelic polymorphisms by definition, but triallelic and tetraallelic SNPs rarely exist in the human population. SNPs are usually considered biallelic, and three genotypes are possible for each SNP locus. They are coded as 0 (homozygous for the wild type), 2 (heterozygote), and 1 (homozygous for mutants) in this study.

Two neighbouring SNP loci of the missing target are considered in the Simpute method. Haplotypes formed by the consecutive pair of loci are constructed and the estimated haplotype probabilities are combined with the LD information from either side of the missing SNP to predict the missing SNP genotype.

### 2.1. Estimate the Population Proportion of Haplotypes

We first considered genotypes at two loci. The counts of all genotype combinations are summarized in Table 3.

In Table 3, there are nine genotype combinations. The haplotypes for eight of them can be clearly resolved, while those of the *N*_1,1_ could be either ab/AB or aB/Ab. The proportion of the four haplotypes can be estimated as follows:

(1) p(ab)=2×N0,0+N0,1+N1,0+X1×N1,12×NP,Q,p(aB)=2×N0,2+N0,1+N1,2+X2×N1,12×NP,Q,p(Ab)=2×N2,0+N2,1+N1,0+X2×N1,12×NP,Q,p(AB)=2×N2,2+N2,1+N1,2+X1×N1,12×NP,Q,,

where *X*_1_ is the proportion of the phase ab/AB with the observed genotype aAbB, and *X*_2_ is the proportion of the phase aB/Ab.

The initial values for *X*_1_ and *X*_2_ are set to 0.5, and they are iteratively updated to get a more probable estimate. The updating step is

(2) X1=p(ab)×p(AB)p(ab)×p(AB)+p(aB)×p(Ab),X2=p(aB)×p(Ab)p(ab)×p(AB)+p(aB)×p(Ab).

The estimated *X*_1_ and *X*_2_ are then used to calculate the *p*(ab), *p*(aB), *p*(Ab), and *p*(AB) in (1). The 10 iterations will stop for either *X*_1_ or *X*_2_. According to (1) and (2), *X*_1_ or *X*_2_ is a cubic function, solved by the cubic formula. Here we use the iterative method to solve *X*_1_ and *X*_2_. The initial value of both is set to 0.5, where the two phases have the same probability (Table 4).

### 2.2. Linkage Disequilibrium Measurement

We impute the missing genotypes using the LD information and the haplotype probabilities calculated in the previous section. If the LD value between two SNP sites is high, then the two SNPs are close to each other, and there are relatively few recombination events between them. Some measurements are commonly used to evaluate the extent of LD between a pair of SNP sites. Two important pairwise measures of LD are *r* ^2^ and |*D*′| [13–15]. Their range is from 0 to 1, but their interpretation is slightly different. When |*D*′| is equal to 1, *r* ^2^ can be small. For example, when *p*(ab) = 0.9, *p*(aB) = 0.1, *p*(Ab) = 0.1, and *p*(AB) = 0, |*D*′| is equal to 1, the *r* ^2^ value is 0.012. In this paper, *r* ^2^ is derived from the input samples. The |*D*′| and *r* ^2^ can be computed as follows.

The difference between the observed and the expected probability of two loci is measured. The disequilibrium coefficient *D* is expressed as

(3) D=p(ab)−p(a·)×p(·b).

The normalized disequilibrium coefficient is defined as *D*′ = *D*/|*D*|_max⁡_ according the study of Pritchard and Przeworski [14], where

(4) $$\begin{array}{c} D_{\max}=\{\begin{array}{cc} \min\left(p\left(\text{a}\cdot \right)\times p\left(\cdot \text{B}\right),p\left(\text{A}\cdot \right)\times p\left(\cdot \text{b}\right)\right), & \text{if}\;D\geq 0\\ \min\left(p\left(\text{a}\cdot \right)\times p\left(\cdot \text{b}\right),p\left(\text{A}\cdot \right)\times p\left(\cdot \text{B}\right)\right), & \text{if}\;D<0. \end{array} \end{array}$$

The range of the normalized disequilibrium coefficient *D*′ is [−1,1]. *D*′ can be 1 while the *P* value is not significant. That is, when *D*′ is equal to 1, there can still be no association. Hence, we adopt another popular measurement *r* ^2^, where

(5) $$\begin{array}{c} r^{2}=\frac{D^{2}}{p\left(\text{a}\cdot \right)\times p\left(\cdot \text{b}\right)\times p\left(\text{A}\cdot \right)\times p\left(\cdot \text{B}\right)}. \end{array}$$

The *r* ^2^ value between the sites *P* and *Q* is denoted as *r*_*P*,*Q*_^2^.

### 2.3. Imputation Algorithm

Consider three SNP sites *P*, *Q*, and *R* that are in consecutive order. The imputation procedure is as follows.

(1) Use the samples with no missing data at *P*, *Q*, and *R* to calculate the pairwise *r* ^2^ at loci *P*, *Q*, and *R*. If the *r* ^2^ equals zero, it will be set to a minimum value of 10^−5^ to facilitate the following computation.

(2) Because most haplotypes consisting of three loci are rare in the population, and the population proportion cannot be correctly estimated with the limited samples in most studies, we approximate it with the product of haplotype proportion for the three pairs of loci and put the LD measured between the two loci as the weights. The probability for haplotype *h*_1_ *h*_2_ *h*_3_ is approximated as

(6) PP,Q,R(h1h2h3)=PP,Q(h1h2)×rP,Q2×PQ,R(h2h3)×rQ,R2×PP,R(h1h3)×rP,R2,

where *P*_*P*,*Q*_(*h*_1_ *h*_2_), *P*_*Q*,*R*_(*h*_2_ *h*_3_), and *P*_*P*,*R*_(*h*_1_ *h*_3_) are the probabilities of haplotype *h*_1_ *h*_2_ at loci *P*, *Q*, haplotype *h*_2_ *h*_3_ at loci *Q*, *R*, and haplotype *h*_1_ *h*_3_ at loci *P*, *R*. These probabilities were generated by (1). 

(3) Calculate the weighting score of genotype ⊗⊕ at each pair of loci:

(7) $$\begin{array}{c} W_{\left(⊗,⊕\right)}=1-\left|\frac{N_{⊗,\cdot }\times N_{\cdot ,⊕}}{N\times N}-\frac{N_{⊗,⊕}}{N}\right|, \end{array}$$

where ⊗ and ⊕ are the genotypes at the first and the second locus in each pair. If the *W*_(⊗,⊕)_ equals zero, it will be set to a minimum value of 10^−5^ to facilitate the following computation.

(4) Calculate the haplotype pair score

(8) score=(PP,Q,R(h1h2h3)+PP,Q,R(h1′h2′h3′))×N⊗,⊕P,Q×N⊗,∘P,R×N⊕,∘Q,RN⊗,·P,Q×N·,∘P,R×N⊕,·Q,R×W(⊗,⊕)×W(⊗,∘)×W(⊕,∘),

where the probabilities of the haplotype pair *P*_*P*,*Q*,*R*_(*h*_1_ *h*_2_ *h*_3_) and *P*_*P*,*Q*,*R*_(*h*_1_′*h*_2_′*h*_3_′) are calculated by (6), and ⊗, ⊕, ∘ represent the same genotypes (*h*_1_ *h*_1_′), (*h*_2_ *h*_2_′), and (*h*_3_ *h*_3_′) at locus *P*, *Q*, and *R*, respectively. 

(5) Choose from all legitimate haplotype pairs that maximize the score in (8). 

The algorithm also considers the situation when consecutive SNPs are missing. In that case, the two neighbouring loci *P* and *R* of the missing locus *Q* can represent the adjacent two loci on the same side of the *Q*. For example, when there is a long stretch of missing genotypes from SNP 1 to 4 in a specific sample, we can first impute locus 4 with information from locus 5 and 6 and then sequentially fill in all the missing ones. 

### 2.4. Time Complexity

Our algorithm requires the computation complexity at the order of *O*(*mw* + *n*) where *n* is the number of missing SNPs, *w* is the number of the SNP loci with at least one missing entry, and *m* is the number of individual with at least one locus missing. Each sample requires the order of *O*(1) to count each of the 9 genotype and the order of *O*(*mw*) for steps 1 and 2. Hence, the total complexity of the algorithm is *O*(*mw* + *n*).

## 3. Data Description

In this paper, we used two data sets to compare imputation performance. All data sets are based on the individuals included in the HapMap project [16].

### 3.1. SNP-Dense Region on Chromosome 22

The first data set was the testing region adopted from Jung et al. They identified a region with dense SNP distribution and demonstrated their performance with only six SNPs, as annotated in HapMap Phase II, release 22. Those SNPs are rs2213329, rs2227029, rs9610029, rs2213331, rs9619447 and rs743726, and are located from positions 33227611 to 33233156 of chromosome 22. This region was selected for its strong linkage of |*D*′| > 0.7. We used the SNP data of 270 people from HapMap to generate the testing data. The data were randomly selected with missing rates of 5%, 10%, 15%, and 20% from the total of 270 × 6 = 1620 SNPs. We adopted the settings of the missing rates of Jung et al. for comparison purposes. This random procedure was repeated 100 times, and the average error rates were obtained. A more realistic comparison is demonstrated with the other set of random missing studies described in the following section.

### 3.2. Random Missing SNPs from the HapMap Phase III on Chromosome 21

We used samples of HapMap phase III as our testing data. Because some of the software we compared required reference data, we provided samples of HapMap Phase II release 22 as the reference samples; those samples were, thus, excluded in our testing set. SNP loci that are tri-alleic or tetraallelic were excluded in the comparison; Tables 1 and 2 show the proportion of this type of loci in the reference samples (HapMap Phase II release 22) and testing samples (HapMap Phase III specific samples), respectively. The proportion is low and is not crucial for the conclusion. We conducted the experiment on the smallest chromosome to enable easier computation of the less efficient algorithms in the comparison. The results are reported separately for the different ethnic groups because certain interesting differences were observed. 

We generated three sets of testing data from the HapMap Phase III specific samples. The first set was derived by randomly masking the genotypes on chromosome 21, called the *complete set*. Because the error rate of genotype calling is less than 1% [17], the missing rates were 0.1%, 0.5%, 1%, and 5%. Ten randomly missing testing data sets were generated for comparison, and the accuracy was calculated as the average of the 10 repeats. The software used to compare the data set included BIMBAM v0.99, BEAGLE v3.0.3, IMPUTE v0.5.0, MARCH v1.0.16, PLINK, and SNPMSTAT v3.1 and used the system Linux kernel version 2.6 on AMD 64 platform.

Our second test data consisted of numerous regions of only three SNPs on chromosome 21, called the *short input*. At most, two of the three SNPs were permitted to be missing in our random sampling process. The error rates are reported, with the averages of 25 repeats of the random missing procedure for missing rates, as 0.1%, 0.5%, 1%, and 5%.

The algorithm we proposed adopted minimum information to complete the missing gaps, and, hence, it is not designed for all purposes. We show that its performance at the highly linked regions is no worse than the best method previously mentioned. The third set of test data consists of missing SNPs with strong linkage (*r* ^2^ > 0.9) to either one of their adjacent SNPs, called *high LD*. The advantage acquired at the highly linked regions is the most important aspect of Simpute and is why Simpute is the most helpful program in global genome sequencing projects. The error rates are reported from the average of 100 repeats of the random missing procedure for the missing rates at 0.1%, 0.5%, 1%, and 5%. 

## 4. Results

**Figure 1:**
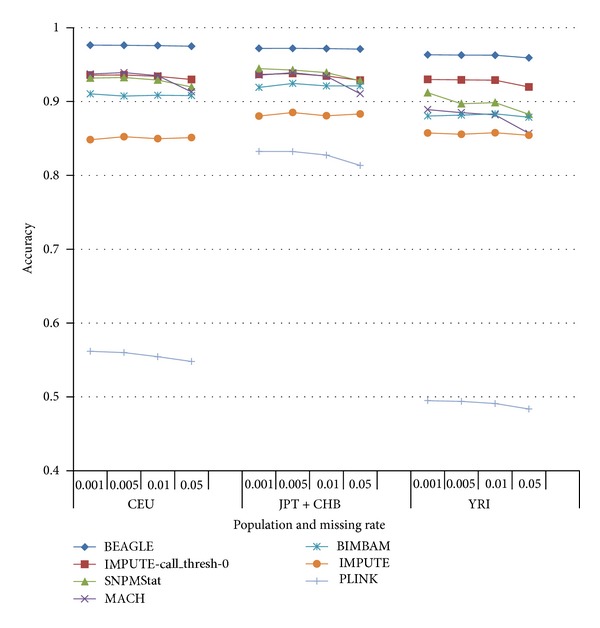
Imputation accuracy compared across BEAGLE, IMPUTE, BIMBAM, SNPMStat, MACH, and plink using the *complete set*. The curve with IMPUTE-call_thresh-0 stands for the best setting (call thresh = 0) we found for Impute rather than the default setting. Accuracy = number of correctly imputed entries/number of missing entries ∗100%.

**Figure 2:**
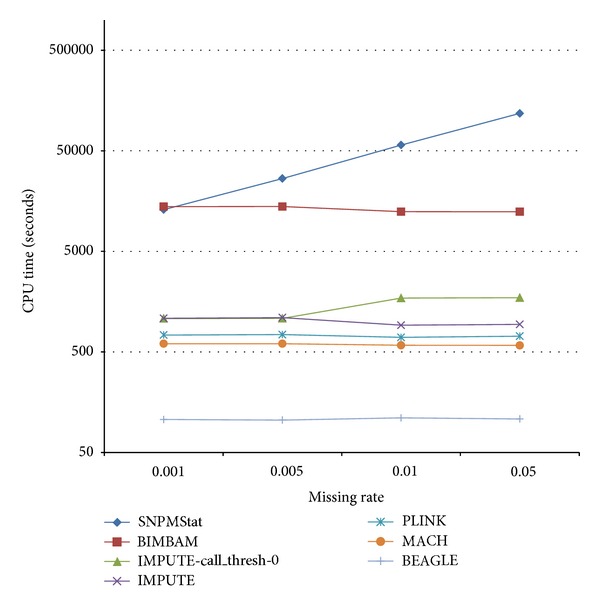
CPU Time.

We used samples from HapMap Phase II release 22 as the reference data set, which is required by BEAGLE, BIMBAM, MACH, SNPMStat, IMPUTE, and plink. Because of the intractable computation load of SNPMStat and IMPUTE, we divided the chromosome into segment of 10,000 SNPs for the inputs. Because SNPMStat requires substantial CPU time, only three repeats were performed to derive the average accuracy. All the other programs used 10 repeats to obtain the averages. The results from the *complete set* are shown in Figure 1. BEAGLE gives the best overall accuracy and is also the fastest on our benchmark platform CentOS 5.3 under the VNWare ESX 4i in Figure 2. The following comparisons only address the differences between Simpute and BEAGLE.

The results from the SNP-dense region of chromosome 22 in Jung's study are shown in Table 5. The error rates from the Jung et al. study are copied directly from their report because we did not implement their algorithm. It appears that Simpute has a strong advantage in the SNP-dense regions. Although BEAGLE used the same HapMap samples as reference samples and used all six SNPs together in their complicated algorithms, it still has slightly higher error rates, and the contrast is strong at the lower missing rates.

To understand the relation between the information fed into each method and the power each method gains, we first assessed the sets of three SNPs on chromosome 21. This provided limited information, and the error rates for Simpute and BEAGLE are shown in Table 6. The missing rates were set as 0.1%, 0.5%, 1%, and 5% to better match the actual applications. Because the data are artificial and require repeated initiation processes for BEAGLE to process all the short regions, extensive computation time is required for BEAGLE to process all the data. Hence, comparing the computation time is not feasible, and it is difficult to run the entire set of simulations on all three ethnic groups. We only reported the results for Group CEU with 25 repeats of the random missing procedure, as shown in Table 6. The error rate of Simpute is approximately the same as that of BEAGLE and matches our expectations.

Tables 7, 8, and 9 show the evaluation of Simpute and BEAGLE using the *high LD* testing data on chromosome 21. The default setting of BEAGLE used the same 270 people from HapMap Phase II as the reference data. In contrast, Simpute used the two neighboring SNPs of the missing one. The error counts are the averages of 100 repeats of the random missing procedure. BEAGLE performed better than Simpute but the difference is negligible when the missing rate is low. In addition, BEAGLE requires substantially more processing time. 

## 5. Conclusion and Discussion 

In this study we developed a simple strategy to impute missing genotypes for SNPs that have a high resolution. Our method requires only two neighbouring loci of a missing SNP. Furthermore, we show in our study that for highly linked loci, our algorithm has comparable performance to BEAGLE, a system that incorporates data from various sources of information, as has been suggested in recent studies. These sources of information include reference samples and long-range LD. 

The algorithm we introduced in our study has a complexity of *O*(*mw* + *n*), where *n*  is the number of missing SNPs, *w* is the number of the positions of the missing SNPs, and *m* is the sample size. Because of the design of our algorithm, and the reduction of the prerequisite input incorporated into the imputation algorithm, we were able to significantly reduce the computation time. 

Although Simpute is unable to outperform most software for general purposes, it has shown its potential for specific purposes. With the current trend of mass parallel-sequencing technologies, SNPs will soon be discovered with ease, without requiring the use of predefined positions for their detection. Furthermore, the availability of samples will accumulate in the following few years. Thus, it is expected that most SNPs will be highly linked in samples of moderate size.

Simpute has a strong advantage over more complicated algorithms that use high LD regions. Moreover, it demonstrates a distinct advantage in efficiency when handling large data sets. This efficiency is of great benefit to genome centers, which have increasing demands in the number of personal genomes that must be sequenced and analyzed through a real-time system.

## Figures and Tables

**Table 1:** The nonbiallelic loci proportion in the HapMap phase II release 22.

Population	Individuals	SNPs	Nonbiallelic
CEU	90	48217	1.69%
JPT + CHB	90	50053	1.81%
YRI	90	48541	1.60%

**Table 2:** The non-bi-allelic loci proportion in the HapMap phase III.

Population	Individuals	SNPs	Non-bi-allelic
CEU	80	19250	0.39%
JPT + CHB	77	17286	0.21%
YRI	80	20198	0.21%

**Table 3:** A 3 × 3 contingency table for the genotypes at two consecutive loci. A and a are the two alleles in locus 1 while B and b are the two alleles in locus 2.

	0 (bb)	1 (bB)	2 (BB)	Total
0 (aa)	*N*_0,0_	*N*_0,1_	*N*_0,2_	*N*_0,·_
1 (aA)	*N*_1,0_	*N*_1,1_	*N*_1,2_	*N*_1,·_
2 (AA)	*N*_2,0_	*N*_2,1_	*N*_2,2_	*N*_2,·_

Total	*N*_·,0_	*N*_·,1_	*N*_·,2_	*N*_*P*,*Q*_

**Table 4:** Notation for the haplotype probabilities at the two loci.

Locus *P*∖*Q*	b	B	Total
a	*p* (ab)	*p* (aB)	*p* (a·)
A	*p* (Ab)	*p* (AB)	*p* (A·)

Total	*p* (·b)	*p* (·B)	1

**Table 5:** Error rates* for Simpute, BEAGLE, and Jung's method with random missing study on the six SNPs of chromosome 22.

Missing rate/method	Simpute	BEAGLE	Jung's method
5%	1.358%	1.7531%	16.59%
10%	1.8944%	2.1429%	17.82%
15%	3.0207%	3.4132%	20.25%
20%	4.4472%	4.4907%	20.07%

*Error rates = number of error imputed entries/number of missing entries ∗100%.

**Table 6:** The error rates* for random missing SNPs of short input at *r* ^2^ ≥ 0.9 from the HapMap phase III on chromosome 21 of short input for the CEU.

Method/missing rate	Simpute	BEAGLE
0.1%	37.136/483 (7.69%)	38.09/483 (7.89%)
0.5%	188/2412 (7.79%)	183.6364/2412 (7.61%)
1%	378.333/4823 (7.84%)	376.762/4823 (7.81%)
5%	1913.632/24111 (7.94%)	1892.053/24111 (7.84%)

*Error rates = number of error imputed entries/number of missing entries ∗100%.

**Table 7:** Error rates* and computation time for random missing SNPs of high LD for the CEU samples.

Method/missing rate	Simpute		BEAGLE	
	Error rate	Running time (sec)	Error rate	Running time (sec)
0.1%	5.52/483 (1.14%)	12.88	4.49/483 (0.93%)	164.17
0.5%	27.94/2412 (1.16%)	13.09	21.01/2412 (0.87%)	164.82
1%	57.22/4823 (1.19%)	14.07	44.07/4823 (0.91%)	168.47
5%	321.9/24111 (1.33%)	18.24	224.65/24111 (0.974%)	168.69

*Error rates = number of error imputed entries/number of missing entries ∗100%.

**Table 8:** Error rates* and computation time for random missing SNPs of high LD for the CHB + JPT samples.

Method/missing rate	Simpute		BEAGLE	
	Error rate	Running time (sec)	Error rate	Running time (sec)
0.1%	5.15/493 (1.04%)	10.90	4.64/493 (0.94%)	138.40
0.5%	27.29/2463 (1.10%)	11.08	24/2463 (0.97%)	139.79
1%	55.07/4925 (1.11%)	11.77	47.69/4925 (0.97%)	138.96
5%	322.38/24622 (1.31%)	16.113	242.26/24622 (0.98%)	140.96

*Error rates = number of error imputed entries/number of missing entries ∗100%.

**Table 9:** Error rates* and computation time for random missing SNPs of high LD for the YRI samples.

Method/missing rate	Simpute		BEAGLE	
	Error rate	Running time (sec)	Error rate	Running time (sec)
0.1%	2.57/271 (0.95%)	12.42	2.23/271 (0.82%)	187.80
0.5%	13.54/1353 (1.00%)	12.925	11.2/1353 (0.83%)	188.41
1%	27.19/2705 (1.00%)	13.08	22.89/2705 (0.85%)	187.45
5%	161.02/13525 (1.19%)	15.921	119.94/13525 (0.887%)	191.29

*Error rates = number of error imputed entries/number of missing entries ∗100%.
